# Anti-T-lymphocyte globulin improves GvHD-free and relapse-free survival in myelofibrosis after matched related or unrelated donor transplantation

**DOI:** 10.1038/s41409-024-02291-6

**Published:** 2024-05-21

**Authors:** Kristin Rathje, Nico Gagelmann, Rachel B. Salit, Thomas Schroeder, Carmelo Gurnari, Simona Pagliuca, Victoria Panagiota, Christina Rautenberg, Bruno Cassinat, Felicitas Thol, Marie Robin, Sofia Oechsler, Michael Heuser, Marie-Thérèse Rubio, Jaroslaw P. Maciejewski, Hans Christian Reinhardt, Bart L. Scott, Nicolaus Kröger

**Affiliations:** 1 Department of Stem Cell Transplantation, University Medical Center Hamburg-Eppendorf, Hamburg, Germany; 2 Fred Hutchinson Cancer Research Center, Seattle, WA USA; 3 Department of Hematology and Stem Cell Transplantation, West German Cancer Center, University Hospital of Essen, Essen, Germany; 4 Translational Hematology and Oncology Research Department, Taussig Cancer Center, Cleveland Clinic, Cleveland, OH USA; 5 Department of Biomedicine and Prevention, Tor Vergata University of Rome, Rome, Italy; 6 Department of Hematology, Nancy University Hospital, and UMR 7365, University of Lorraine, Vandoeuvre-lès-Nancy, France; 7 Department of Hematology, Hemostasis, Oncology and Stem Cell Transplantation, Hannover Medical School, Hannover, Germany; 8 APHP, Laboratoire de biologie cellulaire, Hôpital Saint-Louis, Paris, France; 9 Service d’Hématologie-Greffe, Hôpital Saint-Louis, Assistance Publique Hôpitaux de Paris, Paris, France

**Keywords:** Stem-cell research, Myeloproliferative disease

## Abstract

Acute and chronic graft-versus-host disease (GvHD) are major complications of allogeneic hematopoietic cell transplantation (alloHCT). In vivo T-cell depletion with anti-T-lymphocyte globulin (ATLG) as part of the conditioning regimen prior to alloHCT is frequently used as GvHD prophylaxis, but data on its role in myelofibrosis is scarce. We took advantage of an international collaborative network to investigate the impact of ATLG in myelofibrosis undergoing first alloHCT. We included 707 patients (*n* = 469 ATLG and *n* = 238 non-ATLG prophylaxis). The cumulative incidence of acute GvHD grade II-IV was 30% for the ATLG group vs. 56% for the non-ATLG group (*P* < 0.001). Acute GvHD grade III-IV occurred in 20% vs. 25%, respectively (*P* = 0.01). Incidence of mild-to-severe chronic GvHD was 49% vs. 50% (*P* = 0.52), while ATLG showed significantly lower rates of severe chronic GvHD (7% vs. 18%; *P* = 0.04). GvHD-free and relapse-free survival (GRFS) at 6 years was 45% for the ATLG group vs. 37% for the non-ATLG group (*P* = 0.02), driven by significantly improved GRFS of ATLG in matched related and matched unrelated donors. No significant differences in risk for relapse, non-relapse mortality, and overall survival were observed. Multivariable modeling for GRFS showed a 48% reduced risk of GvHD, relapse, or death when using ATLG.

## Introduction

Myelofibrosis is a *Philadelphia chromosome*-negative myeloproliferative neoplasm (MPN) with a heterogenous clinical phenotype characterized by splenomegaly, progressive anemia, ineffective hematopoiesis, and constitutional symptoms. It can present as a primary disease (primary myelofibrosis, PMF) or evolve from polycythemia vera or essential thrombocythemia (secondary myelofibrosis) [1, 2].

Despite recent improvements in the field of *JAK* inhibitors and novel agents with alternative targets, allogeneic hematopoietic stem cell transplantation (alloHCT) remains the only potentially curative treatment for patients with myelofibrosis to date [2, 3]. However, alloHCT is associated with treatment-related morbidity and mortality, especially in older and comorbid myelofibrosis patients [4, 5]. Acute and chronic graft-versus-host disease (GvHD) are two major life-threatening complications for patients undergoing transplant and they appear to occur more frequently in patients with MPN rather than in those with other hematological malignancies, configuring a field of deep unmet medical needs [6].

In vivo T-cell depletion with rabbit antithymocyte/antilymphocyte globulin as part of the conditioning regimen prior to alloHCT is widely used as an addition to the standard GvHD prophylaxis with a calcineurin inhibitor (cyclosporine or tacrolimus) and either methotrexate or mycophenolate mofetil. Currently, there are two commercial rabbit antithymocyte/antilymphocyte globulin formulations available for clinical practice: antithymocyte globulin (ATG) (thymoglobulin) is generated by immunizing rabbits with human thymocytes whereas antilymphocyte globulin (ATLG) is derived from rabbits vaccinated with the human Jurkat T-cell line. Patient outcomes for the two preparations seem to be similar [7, 8].

Several randomized clinical trials have shown that ATG/ATLG can both prevent incidence and severity of acute and chronic GvHD after alloHCT from unrelated and related donors [9–15]. However, evidence on their role in the setting of myelofibrosis is scarce. The aim of this study was to evaluate the impact of ATLG on clinical outcomes of patients with myelofibrosis undergoing an alloHCT in a large international retrospective study.

## Methods

### Data collection

Patients with PMF, post-polycythemia vera and post-essential thrombocythemia myelofibrosis (post-PV MF and post-ET MF) undergoing first alloHCT between 1992 and 2022 were included in this study. GvHD prophylaxis consisted either of ATLG (Grafalon, Neovii Biotech, Graefelfing, Germany) or non-ATLG-based strategies. Data were collected from 5 European and US academic transplant centers: the University Medical Center Hamburg-Eppendorf (Hamburg, Germany); the West German Cancer Center (Essen, Germany); the Hannover Medical School (Hannover, Germany); the Fred Hutchinson Cancer Research Center (Seattle, WA); and the Leukemia Program, Department of Hematology and Medical Oncology, Cleveland Clinic, (Cleveland, OH). Patients with transformation to secondary acute leukemia at the time of transplant were not included in this study. Detailed information on transplant-, disease- and patient-specific characteristics was collected including the DIPSS score, which was calculated prior to transplantation [16]. Patients gave informed consent and the study is in accordance with the Helsinki declaration.

### Statistical analysis

Main endpoints of this study were GvHD-free and relapse-free survival (GRFS), which was defined as the absence of grade III-IV acute GvHD, severe chronic GVHD, relapse, or death from any cause [17], and acute GvHD.

Further endpoints were chronic GvHD, relapse incidence, non-relapse mortality, and overall survival. Non-relapse mortality was defined as death from any cause other than recurring disease with relapse as a competing event, whereas overall survival was defined as time from alloHCT to latest follow-up or death from any cause. For the outcome of relapse, death without relapse was the competing event. Acute and chronic GvHD were diagnosed and classified according to previously described criteria [18, 19]. Grade II-IV were included to calculate incidence of acute GvHD. For the development of GvHD, relapse and death without relapse were competing risks.

Categorical variables were compared with the Chi-squared method while continuous variables were analyzed using the Mann–Whitney-test. Survival probabilities were estimated by means of the Kaplan-Meier method and probabilities of acute and chronic GvHD, relapse and non-relapse mortality were computed using the cumulative incidence function, accounting for competing risks. To evaluate independent effects on the endpoint of GRFS, a multivariable model using the Cox regression was developed. Additionally, a Dependent Dirichlet Process model for censored data was used in case of potential violations of the ubiquitous proportional hazards assumption. Variables with a *p* value of <0.05 were considered to be significant. Statistical analyses were done with SPSS version 29.0.1 and R statistical software version 4.0.3.

## Results

### Patients

Overall, we identified 707 patients, of whom 469 received ATLG-based GvHD prophylaxis, while 238 received non-ATLG-based GvHD prophylaxis. Within the non-ALTG cohort, 13 patients underwent mismatched related donor transplant and were treated with post-transplant cyclophosphamide. In the ATLG group, patients with a matched related donor were administered 15 or 30 mg/kg ATLG, whereas patients with a matched unrelated donor were given either 30 or 60 mg/kg ATLG. In addition, all patients received either tacrolimus/cyclosporine plus mycophenolate mofetil and/or methotrexate as GvHD prophylaxis. No significant differences were observed between the two groups with regards to median age at alloHCT (58 years for both) nor frequency of driver mutations, year of transplant and disease risk according to DIPSS. Patients in the ATLG and the non-ATLG group differed for other characteristics, including HLA-match, performance status, ruxolitinib exposure prior to HCT, and conditioning intensity. Notably, the most common stem cell source in the ATLG and the non-ATLG cohort was in both cases peripheral blood (98% vs. 95%) and the most frequently used conditioning regimen was busulfan-fludarabine for patients receiving ATLG-based GvHD prophylaxis (60%) and cyclophosphamide-busulfan for the non-ATLG group (55%). Patient characteristics are summarized in Table 1.

**Table 1:** Patient characteristics.

Characteristics	No ATLG (*n* = 238)		ATLG (*n* = 469)		*P*
Age, y, median (range)	58.0	21.6 – 78.9	58.0	18.0 – 75.6	0.93
Patient´s sex, n (%)					0.05
Male	150	63	258	55	
Female	88	37	211	45	
DIPSS, n (%)					0.18
low	14	10	29	6	
intermediate-1	40	28	130	27	
intermediate-2	76	52	239	50	
high	15	10	78	16	
Diagnosis at HCT, n (%)					0.05
PMF	138	58	308	66	
SMF	100	42	161	34	
Donor type, n (%)					<0.001
MRD	83	35	96	21	
MUD	117	49	285	61	
MMRD	12	5	1	<1	
MMUD	26	11	87	19	
Stem cell source, n (%)					0.02
PB	225	95	459	98	
BM	13	5	10	2	
Conditioning intensity, n (%)					<0.001
RIC	83	35	386	82	
MAC	155	65	83	18	
Karnofsky Performance Status, n (%)					<0.001
90–100	149	73	256	56	
<90	55	27	205	44	
Unknown	34		9		
Constitutional Symptoms, n (%)	66	44	232	51	0.19
Unknown	89		24		
Ruxolitinib pre-HCT, n (%)	119	50	192	41	0.04
Splenectomy pre-HCT, n (%)	17	7	33	7	0.83
Driver mutation genotype, n (%)					0.30
CALR	46	19	96	21	
JAK2	141	59	276	59	
MPL	6	3	24	5	
Triple negative	45	19	73	16	
Conditioning regimen, n (%)					<0.001
BuFlu	27	11	280	60	
CyBu	130	55	1	<1	
TBI-based	57	24	44	9	
TreoFlu	1	<1	46	10	
FluMel	21	9	54	12	
FLAMSA	1	<1	33	7	
Other	1	<1	8	2	
Year of transplant, y, median (range)	2014	1997–2022	2013	1992–2021	0.47

### Engraftment and GvHD

Neutrophil engraftment was similar between both groups (*P* = 0.41), showing incidence at 28 days of 95% for the ATLG group vs. 93% for the non-ATLG group. The cumulative incidence of acute GvHD grade II-IV at day +180 was significantly different between the two groups and amounted to 30% (95% CI, 26–34%) for patients receiving ATLG-based GvHD prophylaxis vs. 56% (95% CI, 50–62%) for the non-ATLG cohort (*P* < 0.001; Table 2). Median time to acute GvHD was significantly different between both groups (*P* = 0.009), being 24 days for the ATLG group vs. 32 days for the non-ATLG group. Acute GvHD grade III-IV occurred in 20% vs. 25% in the cohort given or not given ATLG, respectively (*P* = 0.01), with severe acute GvHD grade IV in 5% vs. 10%. No difference in incidence of mild-to-severe chronic GvHD was observed at 6 years, showing 49% (95% CI, 45-54%) for the ATLG group vs. 50% (95% CI, 44–57%) for the non-ATLG group (*P* = 0.52). In contrast to acute GvHD, median time to chronic GvHD was longer for the ATLG group (*P* = 0.05), being 207 days vs. 182 days for patients receiving no ATLG prior to transplant. Absolute rates for severe chronic GvHD were lower for the ATLG group (7%) vs. non-ATLG group (18%; *P* = 0.04).

**Table 2:** Patient outcomes according to ATLG use.

Characteristics	No ATLG (*n* = 238)		ATLG (*n* = 469)		*P*
	%	95% CI	%	95% CI	
Acute GvHD					
Grade II-IV	56	50–62	30	26–34	<0.001
Grade III-IV	25	19–31	20	16–24	0.01
Chronic GvHD					
All grades	50	44–57	49	45–54	0.52
Severe	18	13–23	7	3–11	0.04
Non-relapse mortality					
1-year	19	14–24	20	17–24	0.80
3-year	29	24–35	27	23–31	0.55
6-year relapse	15	12–18	12	8–16	0.62
6-year overall survival	60	54–67	64	59–68	0.53
GRFS					
3-year	47	40–53	53	48–58	0.04
6-year	37	27–46	45	40–50	0.02

### Survival and relapse

**Fig. 1:**
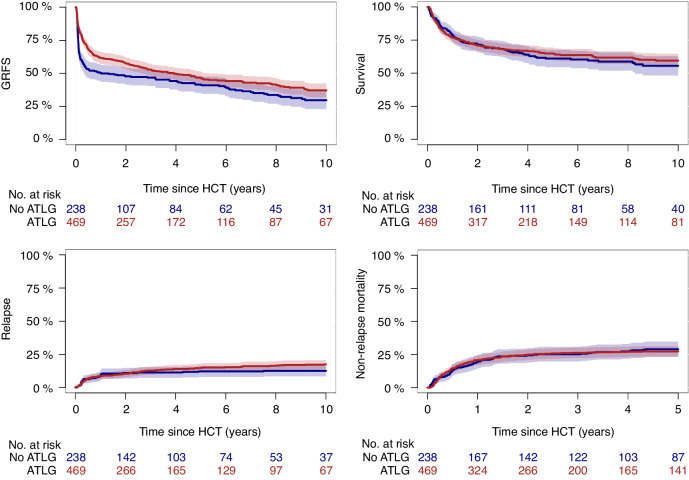
Impact of ATLG on transplant outcomes. GRFS, overall survival, relapse incidence, and non-relapse mortality for the ATLG and non-ATLG cohort.

With a median follow-up of 5.9 years (95% CI, 4.8–6.8 years) for the ATLG group and 6.9 years (95% CI, 5.8–8.2 years) for the non-ATLG group (*P* = 0.07), the 3-year estimates for the composite endpoint of GRFS were 53% (95% CI, 48–58%) for the ATLG group vs. 47% (95% CI, 40–53%) for the non-ATLG group (*P* = 0.04; Fig. 1) and the 6-years estimates of GRFS were 45% (95% CI, 40–50%) vs. 37% (95% CI, 27–46%), respectively (*P* = 0.02). The 6-year estimates for overall survival were 64% (95% CI, 59–68%) for patients receiving an ATLG-based GvHD prophylaxis vs. 60% (95% CI, 54–67%) for the non-ATLG cohort (*P* = 0.53) and the 6-year cumulative incidence of relapse was 12% (95% CI, 8–16%) vs. 15% (95% CI, 12–18%), respectively (*P* = 0.62). The early non-relapse mortality 1 and 3 years after transplant was 20% (95% CI, 17–24%) and 27% (95% CI, 23–31%) for the ATLG group vs. 19% (95% CI, 14–24%) and 29% (95% CI, 24–35%) for the non-ATLG group (*P* = 0.80 and 0.55, respectively).

### Factors on GRFS

In terms of donor type, the use of a mismatched unrelated donor was significantly associated with worse GRFS, showing a hazard ratio of 1.45 (95% confidence interval, 1.13–1.86; *P* = 0.003), whereas other donor types did not impact risk for GvHD, relapse, or death. The intensity of conditioning therapy appeared to influence outcome, showing lower rates of GRFS for myeloablative compared to reduced intensity conditioning (hazards ratio, 1.21; 95% confidence interval, 1.02–1.45; *P* = 0.03). The complete univariable analysis on GRFS including cause-specific hazards is shown in Table 3.

**Table 3:** Univariate analysis on GRFS.

Characteristics	HR	95% CI	*P*
GvHD prophylaxis			
No ATG	Reference		
ATG	0.81	0.68–0.97	0.02
Donor type			
MRD	Reference		
MUD	0.96	0.78–1.17	0.66
MMRD	1.14	0.65–2.01	0.66
MMUD	1.45	1.13–1.86	0.003
Female sex	0.92	0.77–1.10	0.35
Conditioning intensity			
RIC	Reference		
MAC	1.21	1.02–1.45	0.03
Driver mutation genotype			
* CALR*	Reference		
* JAK2*	1.27	1.01–1.60	0.04
* MPL*	0.81	0.48–1.38	0.44
Triple negative	1.18	0.89–1.58	0.25
Ruxolitinib exposure	1.17	0.94–1.48	0.16
Spleen size	1.03	0.95–1.11	0.52
Karnofsky performance status < 90%	1.41	1.15–1.73	<0.001
*ASXL1* mutated	1.54	1.21–1.95	<0.001

**Fig. 2:**
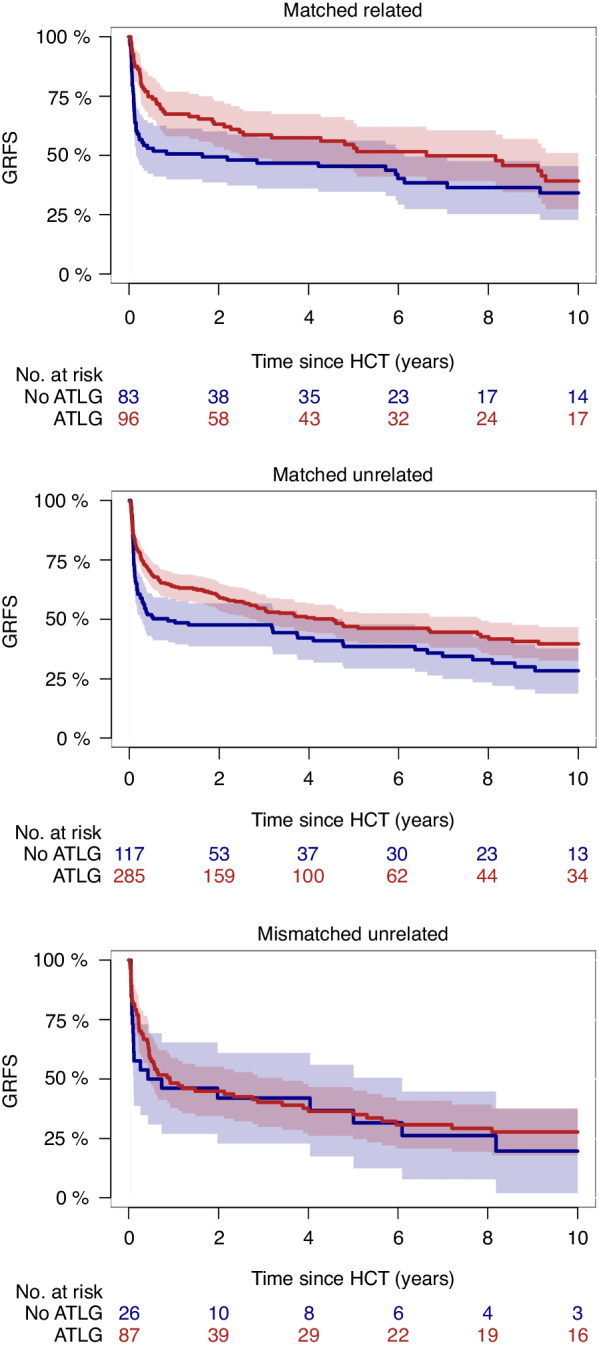
Impact of ATLG in matched related or unrelated donor HCT. GRFS in matched related, matched unrelated, and mismatched unrelated donor transplantation for the ATLG and non-ALTG cohort.

HLA-match did not appear to confound the comparison between ATLG vs. no ATLG, at least in univariable analysis for matched related or unrelated donors, whereas no significant difference in GRFS was found for both groups in the mismatched unrelated setting (Fig. 2). In matched related donor alloHCT, the 6-year estimates of GRFS were 52% (95% CI, 41–62%) for the ATLG group vs. 40% (95% CI, 29–51%) for the non-ATLG group (*P* = 0.05). In matched unrelated donor alloHCT, 6-year GRFS was 46% (95% CI, 32-53%) vs. 39% (95% CI, 29–48%; *P* = 0.03). In mismatched unrelated donor transplants, 6-year GRFS was similar in univariable analysis, showing 31% (95% CI, 21–41%) vs. 32% (95% CI, 12–51%; *P* = 0.70).

### Multivariable analysis

**Fig. 3:**
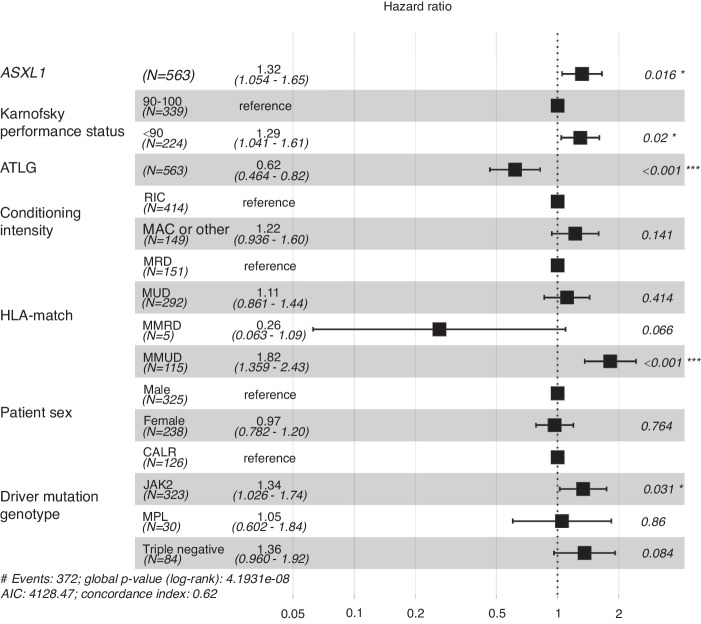
Multivariate analysis on GRFS. Forest plot and corresponding risk ratios of GRFS for clinical and molecular subgroups.

We then developed a multivariable model on GRFS, adjusting for potential confounders and interactions (*ASXL1* and driver mutation status, performance status, HLA-match, conditioning intensity and patient sex). Additionally, a Dependent Dirichlet Process model for censored data was developed, because of violations of the ubiquitous proportional hazards assumption (Fig. 3). The final model was adjusted for a potential interaction of ATLG use and HLA-match, identifying no significant interaction (*P* = 0.10). As a result, the use of ATLG was independently associated with improved GRFS, showing a hazard ratio of 0.62 (95% CI, 0.46–0.82). Other factors associated with worse GRFS were *ASXL1* mutation at time of alloHCT, reduced performance status, mismatched unrelated donor alloHCT, and *JAK2* driver mutation genotype.

## Discussion

To the best of our knowledge, this is the first and largest study evaluating the role of ATLG on transplant outcomes and independent predictors of GRFS in myelofibrosis patients undergoing first alloHCT. We found that the administration of ATLG significantly and independently improved GRFS, with 6-year estimates of 45% for ATLG and 37% for non-ATLG-based GvHD prophylaxis. In our myelofibrosis cohort, this effect was mainly driven by a significant reduction in acute GvHD for ATLG-based GvHD prophylaxis. Incidence of chronic GvHD was similar between both strategies. The effect of ATLG on GRFS was irrespective of HLA-match of patient and donor in multivariable analysis, and most pronounced in patients receiving matched (related or unrelated) donor alloHCT.

Several randomized studies have reported a beneficial impact of ATLG on incidence and severity of GvHD. Kröger et al. conducted a randomized, open-label trial of ATLG added to standard GvHD prophylaxis in 168 patients undergoing matched related donor alloHCT and found significantly lower rates of chronic GvHD and higher GRFS following ATLG without a decrease in incidence of acute GvHD [10]. Other randomized studies among patients receiving alloHCT from unrelated donors have reported that the administration of ATLG resulted in significantly lower rates of acute and chronic GvHD, and with the exception of one study [14], improved GRFS [9]. However, these prospective studies included almost exclusively acute leukemia patients. Of note, the incidence of chronic GvHD in our patients was relatively high, compared to the studies mentioned, whose reported rates were around 30% and resulted lower in the ATLG cohort [9, 10, 14, 15]., but similar to those of other studies including solely myelofibrosis patients [5, 20, 21].

To our knowledge, only one retrospective EBMT study investigated the role of ATLG in 287 myelofibrosis patients and found reduced rates of acute grades II-IV GvHD, while no influence on chronic GvHD, GRFS, overall survival, relapse risk and non-relapse mortality was observed [20]. However, this study also included patients receiving thymoglobulin, which might have affected outcomes, as seen for other previous experiences [7]. Compared to our data, Robin et al. reported lower rates of acute GvHD, but included only patients receiving matched related donor alloHCT. Moreover, due to the lack of data on late acute GvHD, any GvHD occurring after day 100 post-transplant was considered as chronic GvHD in that study. Interestingly, in contrast to our findings, the EBMT analysis showed no significant difference in acute GvHD grade III-IV between the ATLG and non-ATLG cohort.

Another relevant barrier to a successful patient’s outcome remains disease relapse. There have been some contradictory results regarding the incidence of relapse following the administration of ATLG. While few studies have reported an increased risk for relapse in patients receiving ATLG-based GvHD prophylaxis [22, 23], our data, in line with several previous studies[9, 10, 15, 20], suggest no adverse impact of ATLG on absolute recurrence rates of the underlying disease. In addition, incidence of relapse in our cohort was relatively low compared to previously reported rates[20, 24, 25]. Most recently, we were able to show that the risk of relapse was significantly lower in myelofibrosis patients who developed GvHD [26]. These results are in line with an EBMT study showing that the occurrence of GvHD within two years after transplant reduced the risk of relapse in long-term survivors [5]. Stern et al. reported in 2014 that the strength of GvHD and graft-versus-tumor correlation was especially high in patients with myelofibrosis [6]. In our cohort, in spite of significantly lower rates of acute GvHD grade III-IV, we did not find a reduction in non-relapse mortality or an increase in overall survival following the administration of ATLG. This might be due to the fact, that only severe acute GvHD (especially grade IV) seems to be associated with higher risk for death [26].

An essential aspect in alloHCT, which is particularly important for patient counseling, is the selection of the optimal conditioning regimen and intensity prior to transplant. To date, no conditioning intensity has shown to be superior to the other with regards to survival outcomes in myelofibrosis [27, 28]. In this study, the administration of higher intensity conditioning resulted in worse GRFS, although patients treated with myeloablative conditioning were significantly younger (median, 55 years) than patients who received reduced-intensity conditioning (median, 60 years). Concerning the impact of the different conditioning regimens, we showed significantly higher GRFS in patients receiving busulfan-fludarabine-based conditioning. These results are in line with a recent study from CIBMTR favoring busulfan-fludarabine in the setting of myeloablative and reduced-intensity conditioning [29].

In terms of donor choice, the use of mismatched unrelated donors has been reported to be associated with worse survival outcomes [30, 31], while matched related and matched unrelated donor alloHCT showed similar outcomes in several studies [28, 29]. In our cohort, GRFS was significantly worse in individuals transplanted from a mismatched unrelated donor. Notably, we included a minority of 13 patients receiving alloHCT from a mismatched related donor. Although numbers of haploidentical donor alloHCT performed with post-transplant cyclophosphamide have been increasing in the past years, data on its use in myelofibrosis patients remain scarce. Small studies reported contradictory results of haploidentical alloHCT using the post-transplant cyclophosphamide strategy in the setting of myelofibrosis with high relapse and rejection rates [32, 33].

Despite the relatively high GRFS of our cohort compared to other studies [28, 29], GRFS in myelofibrosis remains a significant burden for patients. Compared to other indications such as acute leukemia [17], GRFS appeared similar while events through which this composite endpoint was driven were significantly different. In myelofibrosis, outcome in GRFS is mainly driven by higher incidence of acute and chronic GvHD. Whether outcomes can be improved by new treatment modalities such as the continuation of *JAK* inhibitors throughout the peri-transplant period until stable engraftment is currently being investigated [34–36].

Finally, we acknowledge several limitations that primarily originate from the retrospective nature of our study. We cannot exclude selection bias but aimed to control for it by applying multivariable analysis. Given that patients were included from 5 distinct transplant centers, there is heterogeneity in BMT platforms used. Molecular data other than driver mutations was not available for most individuals. Moreover, we were not able to collect data on timing of ATLG administration nor absolute lymphocyte count, both of which were previously found to influence efficiency of ATLG or transplant outcomes [14, 37].

In conclusion, this collaborative study on myelofibrosis patients undergoing first alloHCT showed significantly improved GRFS following the use of ATLG, both for matched related and matched unrelated donors. This effect was mainly driven by a significant reduction in acute GvHD, whereas incidence of chronic GvHD was similar between the ATLG and non-ATLG group.

## Data Availability

Data can be requested by e-mail to the corresponding author.
